# Sublethal Abamectin as a Population Suppressant: Decoding the Transgenerational Impact on the Asian Citrus Psyllid for Sustainable Management

**DOI:** 10.3390/biology15090683

**Published:** 2026-04-27

**Authors:** Qing Han, Min Xiang, Zhaoquan Yuan, Hui Liu, Biya Gong, Zhongxia Yang

**Affiliations:** 1 Hunan Provincial Key Laboratory for Biology and Control of Plant Diseases and Insect Pests, College of Plant Protection, Hunan Agricultural University, Changsha 410128, Chinazhaoquanyuan@stu.hunau.edu.cn (Z.Y.); 2 Horticultural Research Institute, Hunan Academy of Agricultural Sciences, Changsha 410125, China; minru711@126.com (M.X.); liuhui206218@163.com (H.L.); 3 Yuelushan Laboratory, Changsha 410125, China

**Keywords:** *Diaphorina citri*, abamectin, sublethal effects, transgenerational effects, reproductive potential, IPM

## Abstract

Investigating the effects of sublethal pesticide doses on pest population succession and physiological metabolism is crucial for IPM and resistance delaying. This study evaluated sublethal effects of abamectin on *Diaphorina citri* using two-sex life tables, population modeling, and measurements of hormone levels, energy reserves, and gene expression to reveal its transgenerational impacts. Results showed dose-dependent and transgenerational effects: both F_0_ and F_1_ generations experienced prolonged development and reduced longevity under LC_25_/LC_50_ stress. Life-table parameters (*r*, *λ*, *R*_0_, *GRR*, *T*) declined, and the model predicted a sharp population decrease after 60 days (from 10,357 to 1711 and 372 individuals under LC_25_ and LC_50_, respectively). The findings indicated that abamectin suppresses population growth by delaying development and limiting adult recruitment. Following abamectin treatment, hormone levels (20E and JH) showed dynamic fluctuations with delayed peaks in the treated groups, while energy reserves (glycogen and triglycerides) were generally reduced. Vitellogenin gene expression was mostly suppressed, except for a transient increase in *Vg-1* and *Vg-A1* (LC_25_, day 9), whereas *VgR* was generally up-regulated except in the LC_25_ group. Both LC_25_ and LC_50_ treatments suppressed *D. citri* development and reproduction by disrupting hormone balance and energy metabolism, without inducing hormesis. These findings provide a theoretical basis for optimizing field application strategies and support the use of abamectin in IPM programs to reduce outbreak risk and delay resistance development.

## Simple Summary

*Diaphorina citri* is the primary vector of huanglongbing (HLB). As field populations are frequently exposed to sublethal insecticide residues rather than acute lethal doses, we evaluated the transgenerational effects of abamectin at Lethal Concentration 25% (LC_25_) and Lethal Concentration 50% (LC_50_) on *D. citri* over two consecutive generations. Using age–stage, two-sex life-table, and population projection analyses, we found that abamectin significantly delayed nymphal development, shortened adult longevity, and reduced egg hatching, resulting in sustained declines in key population growth parameters across both generations. These findings indicate that abamectin can exert pronounced transgenerational population suppression under sublethal exposure scenarios, thereby potentially lowering the risk of HLB outbreaks in citrus orchards. The study supports field-oriented dose optimization, application timing, and resistance management strategies within integrated pest management (IPM) programs.

## 1. Introduction

**Figure 1:**
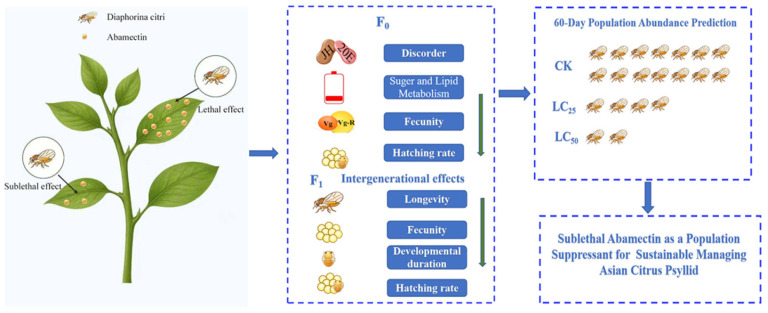
Transgenerational impact of sublethal Abamectin on the *D. citri.*

The efficacy of insecticides is often evaluated based on mortality rates at specific time points. However, under field conditions, due to the spatial and temporal heterogeneity in the absorption, translocation, and degradation of chemicals within leaves and phloem, pests are frequently exposed to a series of varying low concentrations, often existing in a state of “sublethal exposure” rather than experiencing a single high dose with acute lethal effects [1]. Sublethal doses of insecticides can significantly alter the insect development rate, longevity, fecundity, sex ratio, feeding and migratory behavior, as well as learning and memory capabilities, without causing direct mortality [2,3,4,5]. Nevertheless, these sublethal effects are frequently overlooked in traditional efficacy assessments (Figure 1). Consequently, conventional evaluations based solely on mortality rates fail to accurately reflect the true impact of insecticides on pest population growth and their potential for sustained damage [6].

The Asian citrus psyllid, *Diaphorina citri Kuwayama*, is the primary vector of citrus huanglongbing (HLB) and is considered the most devastating pest of citrus crops. It inflicts damage by sucking phloem sap from shoots and young leaves, not only causing direct harm but also, more critically, by acquiring and transmitting the pathogen *Candidatus Liberibacter asiaticus* (CLas). This leads to disease symptoms such as leaf mottling, yellowing, root decline, and ultimately tree death [7,8].

Abamectin is a fermentation product derived from *Streptomyces avermitilis* and belongs to the macrocyclic lactone class of biopesticides. It exhibits both stomach and contact toxicity and is widely used for controlling a variety of agricultural and forestry pests [9,10,11]. The compound primarily acts on glutamate-gated chloride channels (GluCls) and certain gamma-aminobutyric acid (GABA) receptors in invertebrates. By enhancing chloride ion influx, it induces neuronal hyperpolarization, leading to paralysis and eventual death of insects or mites [12]. Due to its unique mode of action and favorable physicochemical properties (abamectin can be used in combination with most pesticides), abamectin serves as a key component in resistance management and insecticide rotation strategies in many cropping systems.

Exposure to abamectin stress, whether at lethal or sublethal doses, disrupts fundamental metabolic processes in insects [13]. Moreover, the stress induced by these doses can be observed in transgenerational offspring even in the absence of further chemical exposure [14,15,16]. For example, the fecundity and wing morph of the F_1_ generation in the brown planthopper and the green peach aphid were significantly altered after parental exposure to abamectin [17,18]. However, some insecticides can induce hormesis at low doses, leading to increased fecundity and population growth. For instance, sublethal doses of abamectin have been shown to cause mild stimulatory effects in the brown planthopper [17]. Therefore, understanding the transgenerational effects of insecticides on pests is crucial for optimizing their application within integrated pest management (IPM) programs.

This study systematically evaluates the comprehensive effects of both sublethal and lethal doses from multiple perspectives, including parental phenotypes, offspring life-table parameters, as well as metabolic and reproductive regulation. It helps clarify why citrus psyllid populations can be effectively suppressed under conventional field application conditions and also reveals the potential risk of low-dose residues in inducing reproductive compensation or population rebound [1].

The related findings not only provide a scientific basis for optimizing field application doses and timing but also offer theoretical support for shifting the control strategy from “rapid individual mortality” to “long-term suppression of reproduction and population growth.” This is of significant importance for reducing the density of citrus psyllids and delaying the evolution of resistance.

## 2. Materials and Methods

### 2.1. Insects and Insecticides

A population of the Asian citrus psyllid, *Diaphorina citri*, was collected in September 2022 from a citrus orchard located in Wantouzhou Village, Yanglin Town, Hengdong County, Hunan Province, China (113°07′ E, 26°59′ N). The population was maintained under laboratory conditions on orange jasmine (*Murraya paniculata*) plants. Rearing conditions were set at 25 ± 1 °C, 60 ± 10% relative humidity, and a photoperiod of L:D = 16:8 h. Host plants were regularly pruned to ensure a continuous supply of fresh flushes for psyllid reproduction. The colony had been maintained indoors for more than 3 years (>18 generations) without exposure to any insecticides.

Abamectin (95.8% purity) was kindly presented by Shenzhen Noposion Agrochemical Co., Ltd. (Shenzhen, Guangdong, China).

### 2.2. Bioassay of Abamectin Toxicity Against D. citri

The toxicity of abamectin to *D. citri* was evaluated using the leaf-dip method under laboratory conditions [19]. Abamectin was first dissolved in acetone to prepare a high-concentration stock solution, which was then diluted with 0.1% Tween-20 aqueous solution to obtain a series of concentrations. Acetone diluted with Tween-20 solution served as the control. Citrus leaves were dipped into the corresponding test solutions for 20 s, allowed to air-dry on plastic film, then wrapped with aluminum foil at the petiole to maintain moisture before being placed into plastic cups.

Thirty 2-day-old adult psyllids, starved for 3 h prior to the experiment, were placed into each cup. The cups were then sealed with ventilated lids. Each treatment included three replicates. Mortality was assessed 48 h after exposure.

Based on the probit analysis of 48 h mortality, the LC_25_ and LC_50_ values of abamectin against 2-day-old adult *D. citri* were determined to be 0.594 mg/L and 2.184 mg/L, respectively. Detailed bioassay results are provided in Supplementary Table S2.

### 2.3. Effects of Abamectin Stress on the Biological Characteristics of D. citri

Two-day-old adult psyllids were exposed to citrus leaves treated with abamectin at either LC_25_ or LC_50_ concentrations. Control insects received leaves treated with acetone only, following the same treatment procedure described in Section 2.2. For each treatment group, 30 pairs of adults were subsequently used in the experiments.

All experiments were performed using specialized insect-rearing tubes. Each tube contained a red mandarin (*Citrus reticulata*) seedling with 4–6 leaves inserted at the base. The tubes were maintained in a climate-controlled incubator set at 25 ± 1 °C, 65 ± 5% relative humidity, and a 16 h:8 h light:dark photoperiod. Adult survival and fecundity were monitored daily. Following each 24 h oviposition period, the seedlings were removed, appropriately labeled, and replaced with fresh ones. Egg hatchability was recorded, and any deceased males were promptly replaced with live counterparts to ensure continuous mating availability until the death of all adult insects.

Eggs laid during the peak oviposition period of the F_0_ generation were collected for further experiments. After hatching, 120 first-instar nymphs were individually transferred to new seedlings and reared in isolation. The developmental duration and survival of each nymph instar were assessed at 24 h intervals. Upon adult emergence, one male and one female were paired in the same rearing tube, and their fecundity as well as egg hatchability were recorded daily until all adults had died.

### 2.4. Effects of Abamectin Stress on Reproduction of D. citri

#### 2.4.1. Determination of JH and 20E Titers

Following exposure to sublethal and lethal concentrations of abamectin, adult *D. citri* were sampled at 1, 3, 5, 7, and 9 days post-treatment, with 30 adults collected per treatment and time point. The samples were homogenized on ice in a 1:9 (*w*/*v*) ratio of body to PBS and then centrifuged at 5000× *g* for 10 min at 4 °C. The resulting supernatant was carefully collected for subsequent hormonal analysis. Juvenile hormone (JH) and 20-hydroxyecdysone (20E) titers were quantified using commercial enzyme-linked immunosorbent assay (ELISA) kits (Fankew, Shanghai, China), strictly according to the manufacturer’s protocols.

#### 2.4.2. Measurement of Glycogen and Triglyceride Contents

At 1, 3, 5, 7, and 9 days post-exposure, 30 adults were collected from each treatment group. The samples were homogenized on ice in a 1:1 (*w*/*v*) ratio of body to extraction buffer and centrifuged at 8000× *g* for 10 min at 4 °C. The resulting supernatant was collected, and the contents of triglyceride and glycogen were determined using commercial assay kits (Solarbio, Beijing, China) in strict accordance with the manufacturer’s instructions.

#### 2.4.3. Analysis of Vitellogenin-Related Gene Expression

Following exposure to sublethal and lethal concentrations of abamectin, adults were sampled at 5, 7, and 9 days post-treatment, with seven individuals collected per treatment group at each time point. The total RNA was isolated using the TRIzol reagent (Invitrogen, Carlsbad, CA, USA) according to the manufacturer’s instructions. First-strand cDNA was synthesized using HiScript^®^ II Q RT SuperMix for qPCR (+gDNA wiper) (Vazyme, Nanjing, China). Quantitative real-time PCR (qPCR) was performed using a Thermo Fisher QuantStudio™ 1 Real-Time PCR System (Thermo Fisher Scientific, Waltham, MA, USA). The β-actin gene (GenBank: DQ675553) was used as the internal reference gene. Gene expression levels were calculated using the 2^−ΔΔCt^ method. All primer sequences and detailed reaction conditions are provided in Supplementary Table S1.

### 2.5. Data Analysis

Probit analysis was performed using statistical software to determine the dose–effect relationship and to calculate the LC_25_ and LC_50_ values of abamectin against *D. citri.* Prior to comparative analysis, all data were checked for normality using the Shapiro–Wilk test and for homogeneity of variances using Levene’s test; datasets meeting the assumptions (*p* > 0.05 for both tests) were retained for further analysis. Differences among the three treatment groups were evaluated by one-way analysis of variance (ANOVA). Where ANOVA indicated significant effects, followed by pairwise *t*-tests (*p* < 0.05) for multiple comparisons. All results are expressed as the means ± standard error (SE). Age–stage, two-sex life-table parameters—including survival, developmental duration, and fecundity of *D. citri* under abamectin exposure—were analyzed with the TWOSEX-MSChart software (version 2.00.2823) [20,21,22,23]. All figures were generated using GraphPad Prism 8 (version 8.4.3) for graphical presentation of the data.

## 3. Results

### 3.1. Effects of Abamectin Stress on Reproduction and Longevity of D. citri

**Figure 2:**
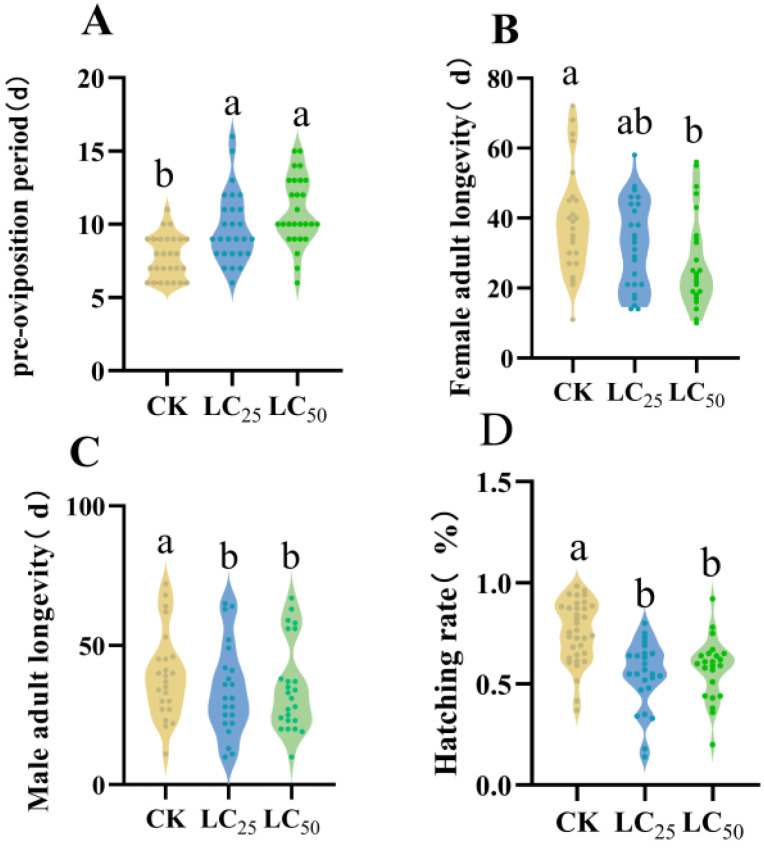
The abamectin stress on parental *D. citri.* Note: (**A**) the pre-oviposition period of female adults; (**B**) the longevity of female adults; (**C**) adult male longevity; and (**D**) the egg hatching rate. (Mean ± SD; the different letters in the figure indicate the significant differences; *p* < 0.05; one-way ANOVA with Duncan’s test).

Compared to the control (CK) group, exposure to abamectin significantly altered key biological traits of *D. citri.* The adult pre-oviposition period was notably prolonged under abamectin stress. While no statistically significant difference was observed between the LC_25_ and LC_50_ treatments, the pre-oviposition period exhibited a dose-dependent lengthening trend: 7.63 d in CK, 10.00 d in LC_25_, and 10.89 d in LC_50_ (Figure 2A).

Abamectin exposure also significantly reduced the longevity of both female and male adults (Figure 2B,C). Additionally, egg hatchability was markedly lower in treated groups compared with the control (Figure 2D).

Collectively, these results demonstrate that abamectin exposure substantially impaired the reproductive performance of the F_0_ generation of *D. citri.*

### 3.2. Effects of Abamectin Stress on the Biological Fitness of Offspring of D. citri

In the F_1_ generation, exposure to abamectin significantly extended the developmental duration of *D. citri*, with the effect progressively magnifying at higher insecticide concentrations and in later nymphal instars. Under LC_25_ and LC_50_ treatments, the developmental duration of first-instar nymphs was prolonged by 0.57 d and 1.05 d under LC_25_ and LC_50_ treatments respectively, compared to the control. This prolongation was sustained from the second through the fourth instar. Notably, in fifth-instar nymphs, developmental duration under the LC_50_ treatment was extended by 1.12 d relative to the LC_25_ group and by 2.08 d relative to the control.

Although adult longevity did not differ significantly between the LC_25_ and LC_50_ groups, both treatments resulted in substantially shorter lifespans than the control reductions of 4.43 d and 2.00 d, respectively. Consequently, the total longevity (from nymph to adult death) was reduced by 4.29 d in the LC_25_ group and by 2.44 d in the LC_50_ group (Table 1).

These findings demonstrate that abamectin, even at sublethal concentrations, induces marked transgenerational inhibition in *D. citri*, primarily by delaying development and shortening the adult lifespan. The effects exhibited a clear dose-dependent pattern and a cumulatively intensifying trend.

**Figure 3:**
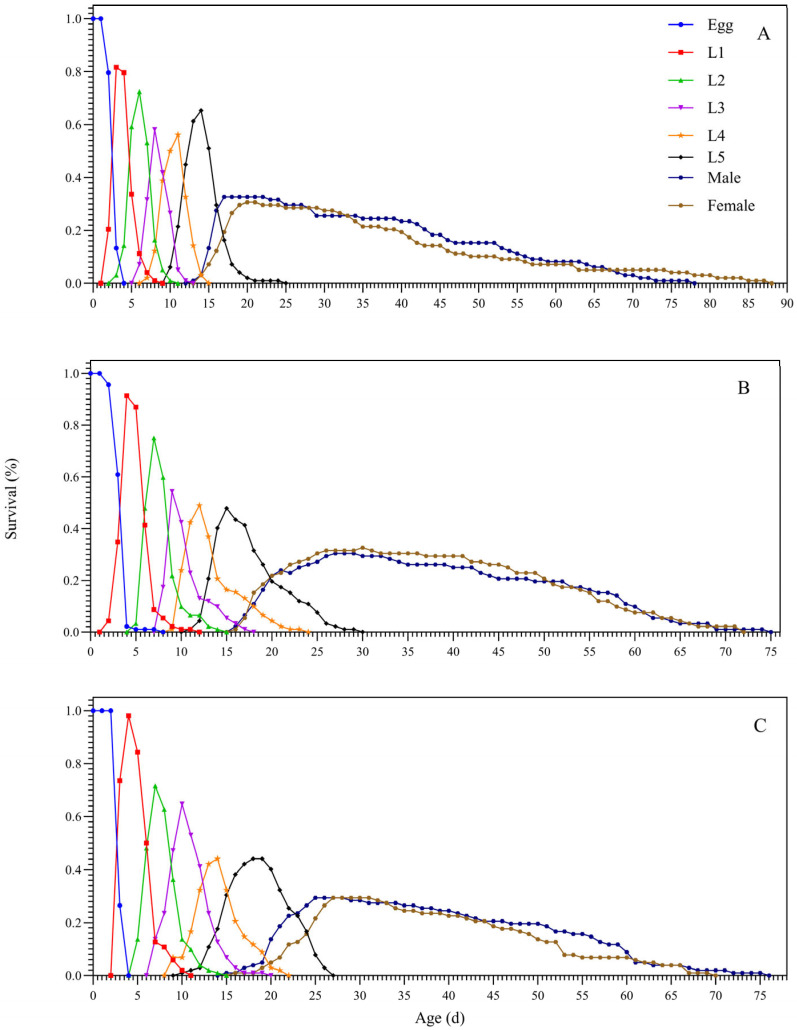
Age–instar survival rate of offspring of *D. citri* after sublethal and lethal concentrations of abamectin treatment. Note: (**A**) CK; (**B**) LC_25_; (**C**) LC_50._

The age–stage-specific survival rates of the F_1_ generation of *D. citri* are presented in Figure 3. Considerable developmental plasticity among individuals led to a substantial overlap in the survival curves across nymphal stages. Compared to the LC_25_ and control (CK) groups, nymphal survival was significantly lower in the LC_50_ treatment. Furthermore, the developmental duration was markedly prolonged, and the total longevity was significantly shortened under LC_50_ exposure. These findings indicate that both sublethal and lethal doses of abamectin not only delay developmental progression but also reduce survival and compress the life cycle of the F_1_ generation of *D. citri.*

Table 2 summarizes the effects of different abamectin concentrations on the population parameters of *D. citri.* Both the intrinsic rate of increase (*r*) and the finite rate of increase (*λ*) varied significantly across treatments, with the lethal dose (LC_50_) exerting a more pronounced negative impact on the offspring’s reproductive performance. Accordingly, the net reproductive rate (*R*_0_) and the gross reproductive rate (*GRR*) in the LC_50_ group were notably lower than those in the control and LC_25_ groups.

Furthermore, abamectin exposure shortened the mean generation time (*T*) of the offspring population values, which were 39.78 d in the control, 36.82 d in the LC_25_ group, and 31.95 d in the LC_50_ group.

Together, these results demonstrate that abamectin suppresses the population growth potential of *D. citri* by simultaneously reducing reproductive output and accelerating generational turnover.

**Figure 4:**
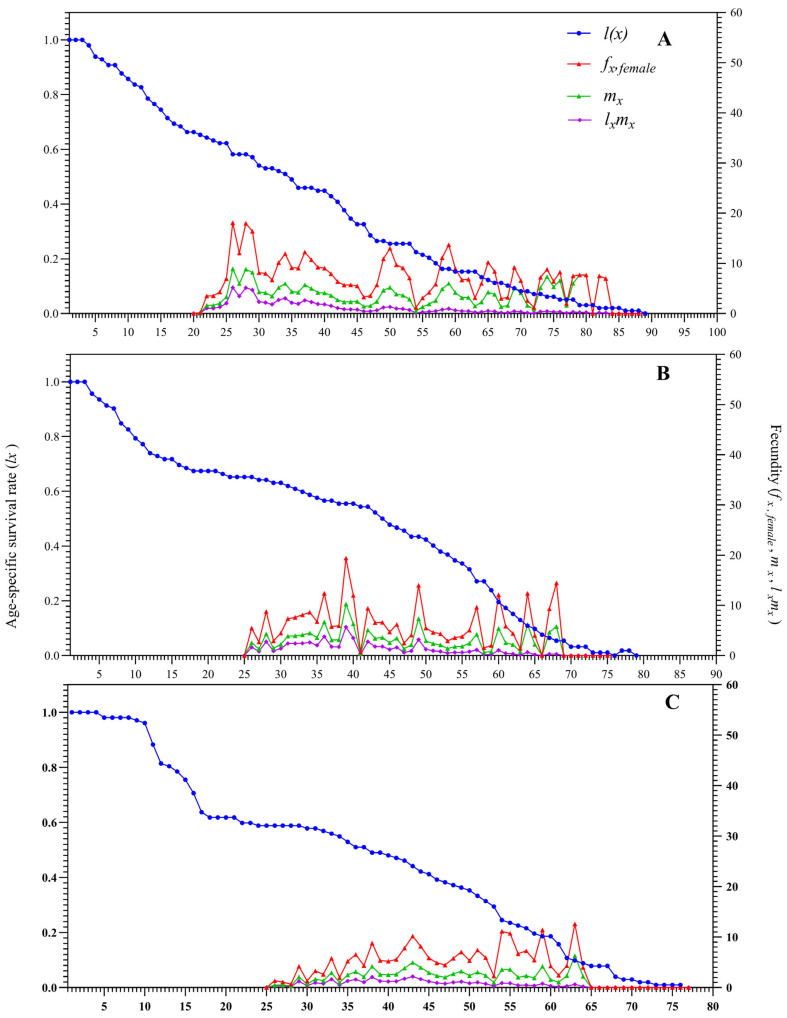
The sublethal and lethal concentrations of abamectin stress on the age-specific survival rate (*l_x_*), female fecundity (*f_x_,_female_*), age-specific fecundity (*m_x_*), and age-specific net reproductive rate (*l_x_m_x_*) of the F_1_ generation of *D. citri*. Note: (**A**) CK; (**B**) LC_25_; and (**C**) LC_50_.

The female age-specific fecundity (*f_x_,_female_*), age-specific survival rate (*l_x_*), age-specific fecundity (*m_x_*), and age-specific net maternity (*l_x_m_x_*) of the offspring population were presented in Figure 4. Abamectin exposure markedly delayed the onset of oviposition by approximately 5–6 days relative to the control. The peak daily fecundity of the offspring in the LC_50_ group was significantly lower than that in the LC_25_ and CK groups.

While survival in the control and LC_25_ groups declined gradually over time, the LC_50_ group exhibited a sharp drop in survival—approximately 40—between 10 and 20 d after emergence.

These results demonstrate that abamectin stress shortens the lifespan and impairs the reproductive output of the offspring population of *D. citri.*

**Figure 5:**
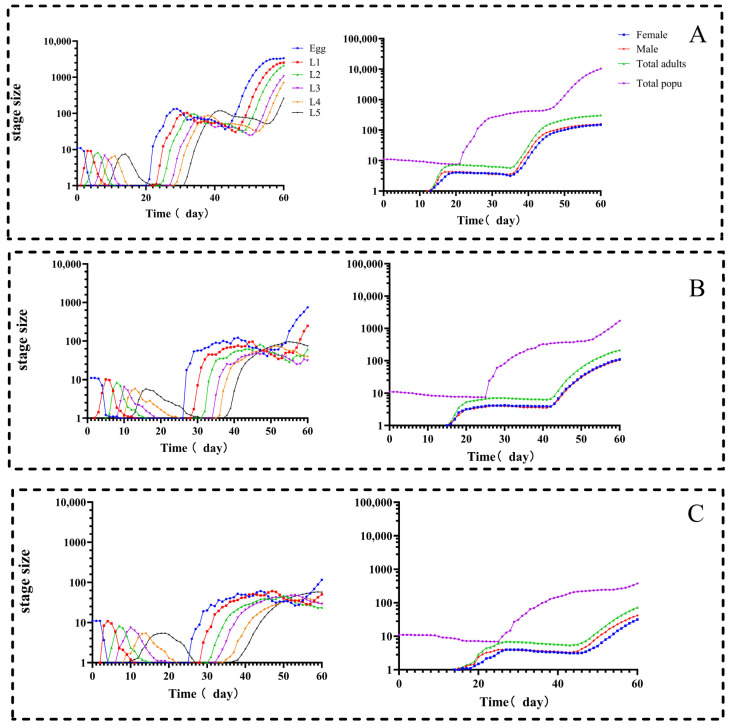
The prediction of population dynamics of *D. citri* in the 60d after sublethal and lethal concentrations of abamectin stress. Note: (**A**) CK; (**B**) LC_25_; and (**C**) LC_50._

The projected population dynamics of *D. citri* over the subsequent 60-day period under abamectin stress were illustrated in Figure 5. The population growth progressed substantially faster in the control group, which exhibited a shorter developmental duration compared to both the LC_25_ and LC_50_ treatment groups. In the control group, fifth-instar nymphs of the first generation completed adult emergence by day 22, whereas emergence was delayed until day 30 in the LC_25_ group and day 28 in the LC_50_ group.

Figure 5 further demonstrates that by day 60, the number of fifth-instar nymphs in the control group peaked at 264 individuals, markedly exceeding those in the LC_25_ (74) and LC_50_ (57) groups. Differences in adult abundance were even more pronounced. The control group contained 308 adults, compared to 212 and 72 adults in the LC_25_ and LC_50_ groups, respectively. Overall, the total population size after 60 days reached 10,357 individuals in the control group, far surpassing those in the LC_25_ (1711 individuals) and LC_50_ (372 individuals) groups.

These findings demonstrate that abamectin can substantially curb the population growth of *D. citri* by retarding development and limiting adult recruitment. Moreover, the inhibitory effect was concentration-dependent, demonstrating that abamectin exerts a sustained and strong regulatory effect at the population level and effectively diminishes the outbreak potential of *D. citri.*

### 3.3. Impact of Abamectin Stress on Hormonal Balance, Energy Metabolism, and Vg Expression in D. citri

**Figure 6:**
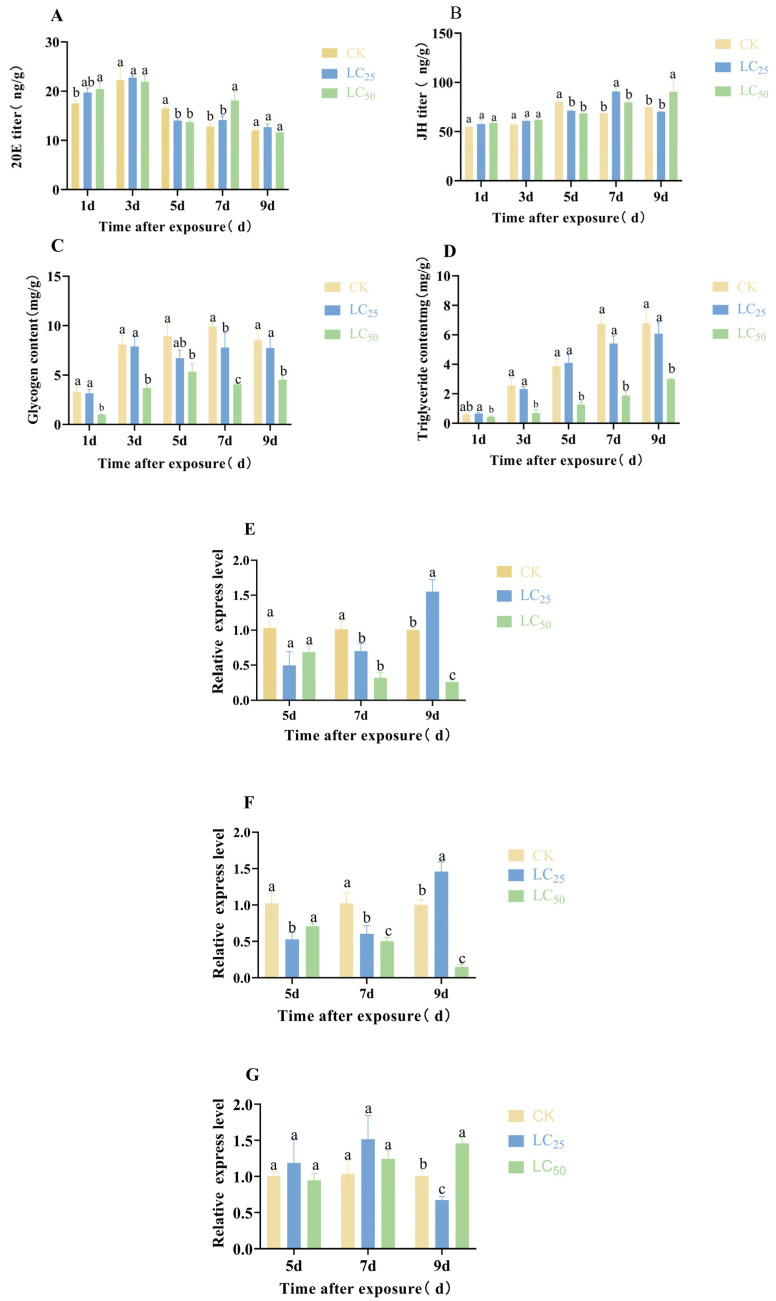

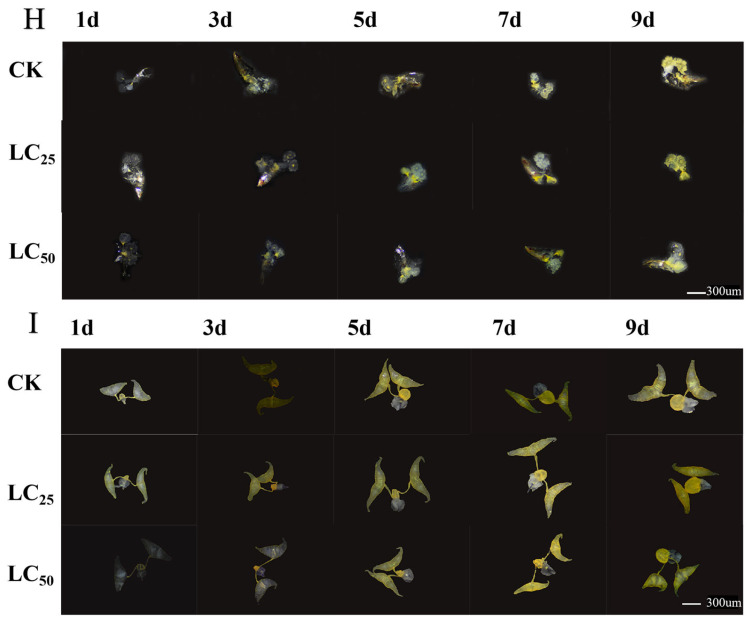
The sublethal and lethal concentrations of abamectin stress on 20-hydroxy ecdysone (20E), juvenile hormone (JH), glycogen and lipid metabolism, and the relative expression levels of *Vg-1, Vg-A1,* and *VgR* in the F_0_ generation of *D. citri.* Note: (**A**) 20-hydroxy ecdysone (20E); (**B**) juvenile hormone (JH); (**C**) glycogen content; (**D**) triglyceride content; (**E**–**G**) relative expression of *Vg-1*, *Vg-A1,* and *VgR*; (**H**) anatomical photograph of the ovary after treatment; and (**I**) anatomical photograph of the testis after treatment. (Mean ± SD. The lowercase letters indicate significant differences among different treatments at the same time point; *p* < 0.05; one-way ANOVA with Duncan’s test).

Abamectin induced significant changes in hormone titers, energy reserves, and the expression of vitellogenesis-related genes in the F_0_ generation of *D. citri* (Figure 6). Notably, the dynamics of 20-hydroxyecdysone (20E) were disrupted. While 20E titers in the LC_50_ group exceeded those of the control on days 1 and 7, a reversal occurred on day 5, with the control group showing the highest levels (Figure 6A).

The temporal peak of juvenile hormone (JH) titers shifted progressively later with increasing abamectin concentration, with the increase occurring at day 5 in the control, day 7 in the LC_25_ group, and day 9 in the LC_50_ group (Figure 6B). Furthermore, both glycogen and triglyceride levels remained significantly lower in the LC_25_ and LC_50_ groups compared to the control at all measured time points from 1 to 9 days post-exposure.

Overall, abamectin treatment downregulated the expression of vitellogenin-related genes *Vg-1* and *Vg-A1*. However, their expression was transiently elevated in the LC_25_ group on day 9. In contrast, *VgR* expression was generally upregulated in treated groups, except in the LC_25_ group at day 9, where it was suppressed.

Collectively, abamectin exposure triggered a cascade of effects in F_0_ *D. citri*: it disrupted the 20E and JH equilibrium, led to a persistent deficit in energy reserves, and ultimately inhibited the expression of vitellogenesis-related genes (*Vg-1*, *Vg-A1*). The transient rebound of these genes in the LC_25_ group at day 9 indicates a degree of physiological compensation under sublethal stress. This interpretation is also supported by the temporal response pattern in the LC_25_ group, in which JH showed an initial increase followed by a decline, whereas 20E continued to rise over time; glycogen tended to stabilize after 3 d, triglyceride as well as *Vg-1* and *Vg-A1* gradually increased, while *VgR* showed a downward trend, suggesting a dynamic adjustment of endocrine, energetic, and reproductive processes under sublethal stress (Figure S1). Notably, the elevated *VgR* expression in the LC_50_ group at the same time point appears to be a feedback response that was insufficient to counteract the overarching suppression of reproductive synthesis.

## 4. Discussion

In practice, factors such as insecticide degradation, plant uptake, and uneven spray coverage often lead to the continuous exposure of *D. citri* to residual or sublethal doses, rather than to a single, acutely lethal dose [11,24,25]. Consequently, conventional assessments based primarily on short-term mortality likely underestimate the long-term regulatory impact of insecticides on pest populations under field conditions.

By integrating age–stage, two-sex life-table analysis with population dynamic simulations, this study systematically assessed the sublethal and lethal impacts of abamectin (LC_25_ and LC_50_) on the individual fecundity and population growth potential of *D. citri.* The results revealed a cumulative inhibitory effect across generations: in the F_0_ generation, abamectin prolonged the pre-oviposition period, shortened adult longevity, lowered egg hatchability, and reduced offspring survival (Figure 2, Figure 3, Figure 4 and Figure 5). In the F_1_ generation, exposure extended nymphal development across all instars, decreased survival rates, and shortened the total lifespan (Table 1, Figure 3). Consequently, key population parameters—including the intrinsic rate of increase (*r*), finite rate of increase (*λ*), net reproductive rate (*R*_0_), and gross reproductive rate (*GRR*)—declined in a dose-dependent manner (Table 2; Figure 4).

These findings demonstrate that the influence of abamectin on *D. citri* extends beyond acute mortality; it exerts sustained, population-level suppression by simultaneously disrupting multiple life-history traits, thereby markedly diminishing the pest’s outbreak potential.

In insects, glycogen and triglycerides serve as essential energy and material reserves sustaining basal metabolism, flight activity, and vitellogenesis [26,27,28]. In this study, abamectin exposure at LC_50_ led to a sustained reduction in both glycogen and triglyceride levels over the 1–9-day period, displaying an overall pattern of “sustained depletion under high stress and localized fluctuation under low stress” (Figure 6). These findings suggest that the abamectin-induced depletion of carbohydrate and lipid reserves likely restricts the energy available for reproductive allocation, thereby impairing ovarian development and ultimately suppressing oviposition capacity.

In insects, juvenile hormone (JH) and 20-hydroxyecdysone (20E) jointly orchestrate growth, development, and reproduction [29,30]. JH maintains high titers during juvenile stages to inhibit metamorphosis and promotes ovarian maturation and vitellogenesis in adults, while 20E primarily triggers molting and metamorphosis and participates in ovarian development and vitellogenesis [31], acting synergistically or antagonistically with JH to finely regulate reproduction [28,29,32,33]. In this study, abamectin disrupted this balance: under LC_50_, 20E titers rose transiently at 1–3 days but fell below control levels thereafter. JH titers exhibited complex temporal dynamics during mid to late stages: at 5 d, treated groups showed lower levels than the control; at 7 d, a transient increase was observed in the LC_25_ group; and a marked elevation in the LC_50_ group at 9 days. These bidirectional, time-dependent fluctuations indicate a significant dysregulation of the JH-20E axis. Importantly, these shifts more likely reflect a stress-induced constraint on energy allocation for reproduction than a true adaptive or hormetic response [34,35].

Vitellogenin (*Vg*) is a well-established biomarker of female reproductive investment in insects [36,37]. In this study, following LC_25_ exposure, relative expression levels of *Vg-1* and *Vg-A1* exhibited partial rebound at certain time points (e.g., 9 d), which coincided with a transient rise in JH titers during the mid-to-late experimental period (Figure 6). Phenotypically, this pattern resembles a localized and limited compensatory endocrine adjustment under mild stress, similar to reports of mild reproductive stimulation induced by sublethal doses of neonicotinoids in some insect pests [38]. This indicates that low-dose abamectin exposure may trigger a restricted compensatory endocrine response. Crucially, however, life-table analyses confirm that this transient molecular and endocrine rebound was insufficient to offset the overarching suppression of development and survival. Consequently, population-level outcomes were consistently characterized by a net reduction in growth potential.

These findings are strongly corroborated by the population projection models. As shown in Figure 5, abamectin treatment resulted in a substantially lower total population size over a 60-day projection compared to the control, with the suppression exhibiting a clear dose dependence (e.g., 10,357 individuals in the control vs. 1711 in LC_25_ and 372 in LC_50_ at 60 days). This demonstrates that the impact of abamectin extends beyond direct mortality to include a sustained, transgenerational suppression of population growth—mediated through inhibited reproduction and delayed development—thereby effectively diminishing the long-term outbreak potential of *D. citri.*

From a field-management perspective, the present results are particularly important because *D. citri* in citrus orchards is often exposed to residual or declining insecticide concentrations rather than uniformly lethal doses under field conditions. In such situations, the significance of sublethal effects lies less in immediate mortality and more in their contribution to long-term population regulation. Even when insecticide exposure does not cause complete mortality, sublethal effects can still delay development, reduce adult reproductive capacity, and slow population growth, which postpones the establishment of the next generation [39,40]. These responses are highly relevant in citrus orchards because they can suppress population resurgence during the interval between insecticide applications, reduce reinfestation on newly emerged flush, and lower cumulative psyllid pressure over time. As a result, sublethal effects provide a more realistic indicator of insecticide performance than mortality alone, especially under field conditions where management success depends not only on rapid knockdown but also on preventing the recovery of the population after treatment. This information is therefore valuable for optimizing spray intervals, improving spray timing based on flush phenology and orchard monitoring, and integrating abamectin into broader IPM programs [41,42]. In addition, by slowing population recovery between applications, sublethal effects may help reduce the need for overly frequent spraying, thereby lowering selection pressure and delaying the development of resistance. Therefore, in practical orchard systems, abamectin should be regarded not only as a compound for direct suppression but also as a tool for longer-term population regulation and the more sustainable management of *D. citri* [43,44].

## 5. Conclusions

This study elucidated the inhibitory and transgenerational effects of abamectin on the citrus psyllid, *Diaphorina citri*, integrating age–stage, two-sex life-table analysis, population dynamics simulation, and molecular regulation. Upon exposure to abamectin, the parental generation (F_0_) showed a significantly prolonged pre-oviposition period, shortened adult female and male longevity, and reduced egg hatchability. These effects extended to the offspring generation (F_1_), resulting in delayed nymphal development, decreased survival rates, and a compressed lifespan. Key demographic parameters—including the intrinsic rate of increase (*r*), finite rate of increase (*λ*), net reproductive rate (*R*_0_), and gross reproductive rate (*GRR*)—were consistently reduced, and 60-day projections indicated a substantial decline in population size. Mechanistically, abamectin disrupted the titers of juvenile hormone (JH) and 20-hydroxyecdysone (20E), induced long-term depletion of energy reserves (glycogen and triglycerides), and broadly suppressed the expression of vitellogenin-related genes, thereby constraining ovarian development. Although transient, low-dose compensatory responses were observed locally, they were insufficient to offset the overall reduction in lifespan and population suppression, with no clear evidence of hormesis. Collectively, these findings support the rotation of abamectin with insecticides of distinct modes of action, adherence to recommended rates and residual intervals, and dynamic optimization of application timing and frequency based on field monitoring (psyllid density and flush phenology). Such an approach may reduce the HLB outbreak risk and delay resistance evolution within integrated pest management (IPM) programs.

## Figures and Tables

**Table 1:** The offspring developmental duration following parental abamectin stress.

Stage	Developmental Time/Longevity (d) (Mean ± SE)					
	CK	n	LC_25_	n	LC_50_	n
Egg	2.93 ± 0.058 b	98	3.62 ± 0.079 a	92	3.26 ± 0.044 a	102
L1	2.37 ± 0.107 b	91	2.94 ± 0.115 a	84	3.42 ± 0.126 a	100
L2	2.42 ± 0.104 b	85	2.77 ± 0.113 a	74	2.7 ± 0.099 a	99
L3	2.01 ± 0.086 b	82	2.33 ± 0.139 a	70	3.4 ± 0.159 a	83
L4	2.53 ± 0.103 b	76	3.42 ± 0.181 a	65	3.48 ± 0.187 a	66
L5	4.3 ± 0.2 c	67	5.16 ± 0.19 b	61	6.38 ± 0.243 a	60
Adult	33.7 ± 2.169 a	67	31.7 ± 1.593 ab	61	29.27 ± 1.592 b	60
Total longevity	39.94 ± 2.288 a	98	37.5 ± 2.401 b	92	35.64 ± 2.092 b	102

Abbreviations: CK, control; LC_25_, lethal concentration of abamectin causing 25% mortality; LC_50_, lethal concentration of abamectin causing 50% mortality; L1–L5, the first to fifth instar nymph stages. Note: Means within a row followed by different lowercase letters are significantly different at *p* < 0.05.

**Table 2:** Effect of sublethal and lethal concentrations of abamectin on the life parameters of the F_1_ generation of *D. citri.*

Population Parameters (Means ± SE)	Conditions		
	CK	LC_25_	LC_50_
Intrinsic rate of increase *r*	0.1333 ± 0.0069 a	0.1116 ± 0.0062 b	0.0089 ± 0.0067 c
Finite rate of increase *λ*	1.1143 ± 0.0079 a	1.1180 ± 0.0070 b	1.0936 ± 0.0074 c
Net reproduction rate *R*_0_	70.74 ± 13.56 a	60.80 ± 11.59 a	35.18 ± 8.82 b
Gross reproductive rate *GRR*	246.37 ± 43.34 a	141.46 ± 29.09 a	96.93 ± 23.34 b
Mean generation time *T* (d)	39.78 ± 0.82 a	36.816 ± 0.97 b	31.95 ± 0.94 c

Abbreviations: CK, control; LC_25_, lethal concentration of abamectin causing 25% mortality; and LC_50_, lethal concentration of abamectin causing 50% mortality. Note: Means within a row followed by different lowercase letters are significantly different at *p* < 0.05 based on the bootstrap test with 100,000 re-samplings.

## Data Availability

The original data presented in the study are openly available in FigShare at https://doi.org/10.6084/m9.figshare.31610326 (accessed on 18 February 2025).

## Supplementary Materials

The following supporting information can be downloaded at https://www.mdpi.com/article/10.3390/biology15090683/s1, Figure S1: Sublethal and lethal concentrations of abamectin stress on 20-hydroxy ecdysone (20E); Juvenile hormone (JH); Glycogen and lipid metabolism; Relative expression levels of *Vg-1, Vg-A1* and *VgR* in F_0_ generation of *D. citri*; Table S1: Primer list used for qRT-PCR, Related to Method details; Table S2: Abamectin resistance in the Asian citrus psyllid.
