# Supplementary material for: Sublethal Abamectin as a Population Suppressant: Decoding the Transgenerational Impact on the Asian Citrus Psyllid for Sustainable Management
Source: Biology (Basel). 2026 Apr 27;15(9):683. doi: 10.3390/biology15090683 (PMC13162577; doi:10.3390/biology15090683)
Supplement: Supplementary file 1 [file biology-15-00683-s001.zip › Supplementary File S1.pdf]

Table S1. Primer list used for qRT-PCR，Related to Method details.

Gene	*Primer Name*	Primer Sequence
Actin	*Actin-F*	CCATCTTGGCTTCTCTGTCTAC
	Actin-R	CATTTGCGGTGAACGATTCC
DcVg-1-like	*DcVg-1-like-qF*	CACCTACTCCTTGTCCTCTA
	DcVg-1-like-qR	GAAAAATCCCCAGAGTCCTT
DcVg-A1-like	*DcVg-A1-like-qF*	CTCCTCAGAAAGTGGAAGTT
	DcVg-A1-like-qR	TTGTTTCCGATGAAGTAGGG
DcVgR	*DcVgR-qF*	AGCAGCTGGATATACATGTG
	DcVgR-qR	CTCCACAGTACTGATTACCG

Table S2. Abamectin resistance in the Asian citrus psyllid

Abamectin 48h	10		5		2.5		1.25		0.625	
	Dead	Total	Dead	Total	Dead	Total	Dead	Total	Dead	Total
1	11	15	10	15	7	15	5	15	4	15
2	12	15	11	15	7	15	6	15	4	15
3	13	15	12	15	8	15	7	15	3	15

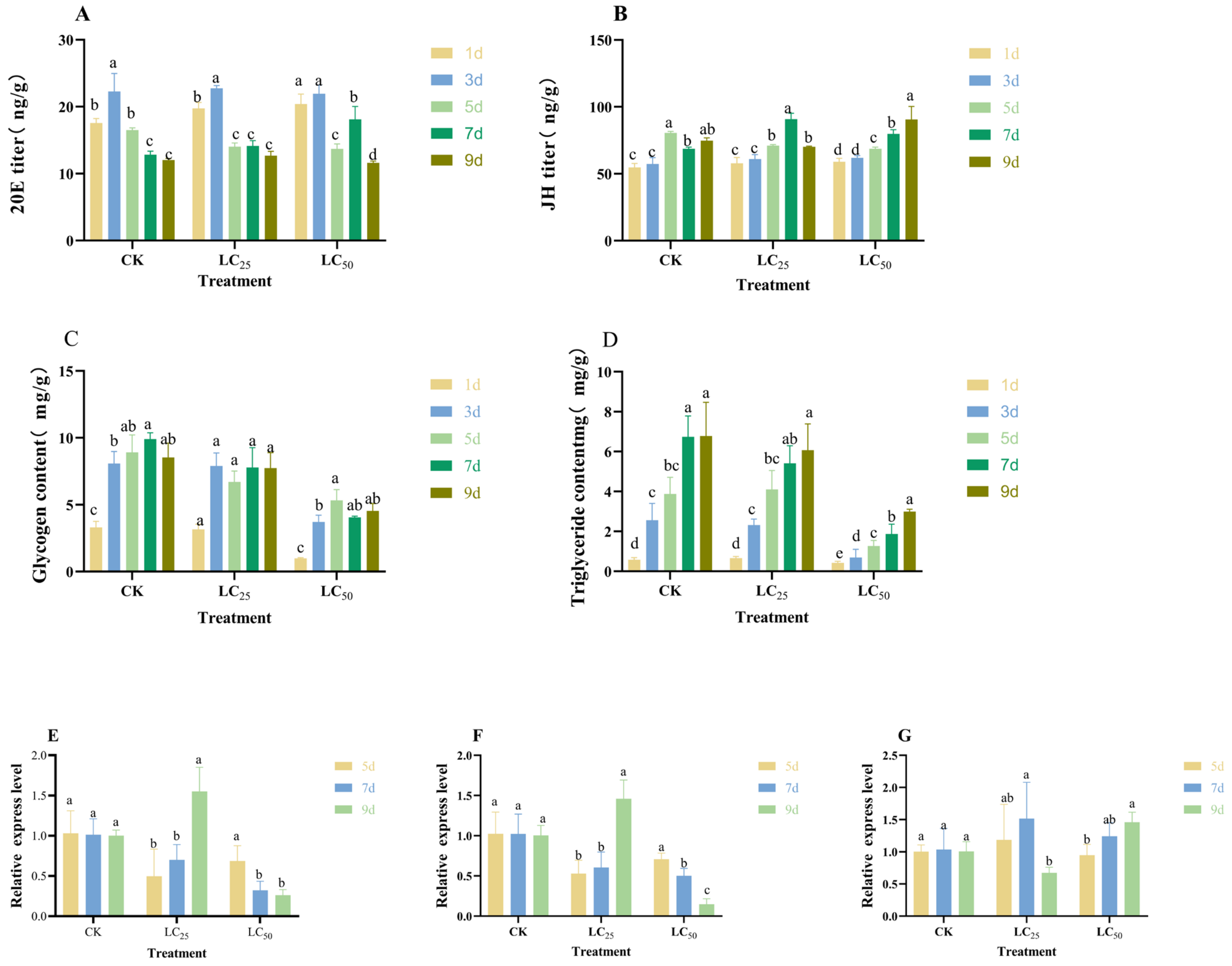

Figure S1. Sublethal and lethal concentrations of abamectin stress on 20-hydroxy ecdysone (20E) ;Juvenile hormone (JH) ;Glycogen and lipid metabolism;Relative expression levels of *Vg-1, Vg-A1* and *VgR* in F_0 generation of *D. citri.*

Note: (A)20-hydroxy ecdysone(20E);(B)Juvenile hormone(JH);(C)Glycogencontent;(D)Triglyceride content;(E-G) Relative expression of *Vg-1,Vg-A1* and *VgR*;

(Mean±SD. Lowercase letters indicate significant differences among different time point at the same treatments; $p < 0.05$; one-way ANOVA with Duncan's test)
